# Clinical Outcomes of Intermediate-Risk Pulmonary Embolism Across a Northeastern Health System: A Multi-Center Retrospective Cohort Study

**DOI:** 10.7759/cureus.15888

**Published:** 2021-06-24

**Authors:** Chuan Jiang, Meng Xie

**Affiliations:** 1 Internal Medicine, Northwell Health, Manhasset, USA; 2 Internal Medicine, Jamaica Hospital Medical Center, Richmond Hill, USA

**Keywords:** pulmonary embolism, fibrinolysis, ultrasound assisted catheter directed thrombolysis, catheter directed thrombolysis, pulmonary hypertension, medical intensive care unit (micu), anticoagulation

## Abstract

Objective: The role of thrombolytic therapy in the management of intermediate-risk pulmonary embolism is controversial. Our objective was to determine clinical outcomes for a population of patients with intermediate-risk pulmonary embolism receiving anticoagulation with and without thrombolytic therapy in a large Northeastern health system.

Design: A retrospective cohort study.

Setting: ICU and non-ICU settings in 8 hospitals.

Patients: Hemodynamically stable patients with intermediate-risk pulmonary embolism.

Interventions: Treatment arms were anticoagulation (AC) alone, AC with low dose intravenous thrombolysis, AC with full-dose intravenous thrombolysis, and AC with ultrasound-assisted, catheter-directed thrombolysis.

Measurements and Main Results: In 257 patients, utilizing a Bonferroni corrected P value cutoff of α = 0.003, our data shows no differences in 7 day or 30 day all-cause mortality (α = 0.37 and α = 0.04, respectively) , hospital length of stay (α = 0.31), 7 or 30 readmission rates (α = 0.97 and α = 0.84, respectively), or any major (α = 0.82) or minor bleeding events (α = 0.007) among the four treatment groups. Use of anticoagulation alone was associated with a lower duration of ICU stay (α < 0.001). There was a significant decrease in the secondary outcome of one year all-cause mortality in favor of full dose and catheter-directed thrombolytic treatment (α = 0.003). Pulmonary artery systolic pressure of > 70 mmHg was associated with increased 7-day mortality (OR 7.79, P = 0.048), and systolic blood pressure < 130 (OR 23.0; P = 0.003) and elevated N-terminal pro-B-type natriuretic peptide > 1400 pg/nl (OR 15.33; P = 0.01) were associated with increased 30- day mortality.

Conclusions: The use of thrombolytic therapy is not associated with a mortality benefit in the first 30 days compared to anticoagulation alone in this patient population and is associated with increased utilization of intensive care unit resources. We advocate for a conservative approach utilizing initial anticoagulation alone in a patient diagnosed with intermediate-risk pulmonary embolism.

## Introduction

Management of intermediate-risk pulmonary embolism (PE), characterized by right ventricular (RV) dysfunction in the absence of systemic hypotension, is a topic of considerable debate. As defined by the European Society of Cardiology European Heart Guidelines, intermediate-risk PE is characterized by signs of RV dysfunction on computed tomography pulmonary angiography (CTPA) or transthoracic echocardiography (TTE) and/or the presence of myocardial necrosis in the absence of systemic hypotension [1]. In contrast, high-risk PE is characterized by the presence of systemic hypotension defined as systolic blood pressure (SBP) < 90mmHg for at least 15 minutes with signs of impaired end-organ perfusion or fall in SBP by at least 40 mmHg for at least 15 minutes with signs of impaired end-organ perfusion. Four major treatment options have been proposed for intermediate-risk pulmonary embolism: standard anticoagulation with unfractionated or low molecular weight heparin, full-dose thrombolytic therapy with 100mg of intravenous alteplase or its equivalent, lower-dose thrombolytic therapy with 50 mg of intravenous alteplase or its equivalent, and ultrasound-assisted catheter-directed thrombolytic therapy, which uses lower doses of alteplase infused locally into the pulmonary artery given over several hours. Several studies have shown the proposed benefits of each thrombolytic therapy compared to anticoagulation alone. For example, the landmark study by Konstantinides showed that patients with intermediate-risk PE who received heparin and 100mg of alteplase over 2 hours had a significantly lower rate of the composite outcome of all-cause mortality and escalation of treatment [2]. The Moderate Pulmonary Embolism Treated With Thrombolysis (MOPETT) trial, the major positive trial favouring lower dose thrombolytic therapy, and the Ultrasound Accelerated Thrombolysis of Pulmonary Embolism (ULTIMA) trial, frequently cited as the main positive trial favouring catheter-directed thrombolytic therapy, have been cited to justify these methods [3,4]. However, these four approaches have never been directly tested head to head in a well-designed, randomized, controlled trial. 

The lack of clarity regarding optimal therapy for intermediate-risk PE is reflected by the differences in the treatment recommendations of two major professional societies. The American Heart Association (AHA) recommends considering peripheral intravenous and catheter-directed thrombolysis at the time of diagnosis [5]. However, the American College of Chest Physicians (ACCP) recommends against the routine use of systemic and catheter-directed thrombolysis. Instead, it favours standard anticoagulation with thrombolytic rescue therapy for worsening respiratory status or hemodynamic instability [6]. This has, in our experience, led to different treatment approaches among cardiologists, pulmonologists, and intensivists. Complicating the matter, only a minority of centres have the expertise necessary in the cardiac catheterization laboratory or interventional radiology suites to consider catheter-directed approaches. We, therefore, undertook a retrospective review of records in Northwell Health, an extensive Northeastern US health system. Northwell consists of 21 hospitals across New York State.

## Materials and methods

Study Design

We conducted a retrospective cohort analysis of all consecutive patients admitted with intermediate-risk PE from January 1, 2012, to February 27, 2017. Northwell maintains a comprehensive database of patient records for outcomes research. International Classification of Diseases (ICD) ICD-9 codes identified patients for pulmonary embolism (I26.0 and all codes within this subset) and ICD-10 codes for acute pulmonary heart disease (415.0 and all codes within this subset). Individual patient records were reviewed for eligibility. Eligible patients were required to have: 1) a diagnosis of PE 2) be at least 18 years old 3) evidence of right ventricular dysfunction, either by echocardiography, CT angiography, or elevated N-terminal pro-B-type natriuretic peptide (NT-proBNP), or evidence of myocardial necrosis by elevation of Troponin T or Troponin I. Exclusion criteria were hemodynamic instability at the time of diagnosis (high-risk pulmonary embolism - defined as systolic blood pressure < 90mmHg or a drop in systolic blood pressure of > 40 mmHg from baseline for a period > 15minutes or hypotension that requires vasopressors or inotropic support), pregnancy, and preexisting anticoagulation therapy. All eligible patients had their relevant demographic, clinical, treatment, and outcome data recorded in a secure database for analysis. Institutional Review Board approval (IRB #: 17-0276) was granted by the Northwell Health IRB as a retrospective chart review. All study protocols were by the Declaration of Helsinki. All data were obtained from the Northwell Health universal electronic medical record system.

The four treatment groups identified were 1) Anticoagulation only (AC), 2) Anticoagulation with low-dose peripherally administered thrombolysis (LDT), 3) Anticoagulation with full-dose peripherally administered thrombolysis (FDT), 4) Anticoagulation with ultrasound-assisted, catheter-directed thrombolysis (US-CDT). In this study, all patients who received peripherally administered systemic thrombolysis received alteplase (tPA, tissue plasminogen activator). For patients prescribed LDT, alteplase was ordered to be infused at 50mg with an initial loading dose of 10mg, followed by an infusion of 40mg over the next two hours in conjunction with anticoagulation. For patients prescribed FDT, alteplase was infused to a total dose of 100mg, with an initial loading dose of 10mg, followed by an infusion of 90mg over the next two hours in conjunction with anticoagulation. The Northwell Health System centres capable of performing US-CDT utilized the EkoSonic® Endovascular System (EKOS, Bothwell, Washington) proprietary technology. This procedure involves placing a catheter into the main pulmonary artery or both pulmonary arteries via common femoral or internal jugular venipuncture and infusing alteplase simultaneously to deliver ultrasonic energy to provide mechanical disruption of the thrombus [7]. The infusion time and dosage of thrombolytics were at the discretion of the physician performing the procedure.

Outcomes

The primary outcomes for this study were seven days and 30-day all-cause mortality from the time of PE diagnosis. Secondary outcomes include hemodynamic instability that developed after initiation of prescribed treatment, one-year mortality from time of PE diagnosis, rate of intensive care unit (ICU) admission for the patients who were initially triaged to a non-ICU setting, length of ICU stay, length of hospital stay, seven days readmission rate, and 30-day readmission rate. Additional outcome analysis was performed among the patients in the AC only group to ascertain if certain clinical risk factors (age, gender, severity of RV dysfunction, hemodynamic data, and echocardiographic indices) are associated with a higher risk of clinically meaningful outcomes.

The primary safety outcomes were incident rates of major bleeding, minor bleeding, hemodynamic instability, need for mechanical ventilation, surgical embolectomy or catheter thrombus fragmentation, and need for vena cava interruption following initiation of prescribed treatment for rescue therapy. Major bleeding was defined according to the International Society on Thrombosis and Haemostasis [8] and entails 1) Fatal bleeding, 2) Symptomatic bleeding in a critical area or organ such as intracranial, intraspinal, intraocular, retroperitoneal, intraarticular, pericardial, or intramuscular with compartment syndrome, 3) Bleeding causing a fall of haemoglobin of 2g/dL or more or leading to a transfusion of two or more units of packed red blood cells. Minor bleeding includes any bleeding episode that does not meet the criteria outlined above.

Statistical Analysis

The English version of SPSS V23.0 (IBM) was used as a database and for statistical analysis. Data are expressed as mean + standard deviation and as a percentage for categorical parameters. Comparison between groups was determined by calculation of the chi-square test for categorical variables. Comparisons between continuous variables were performed by the analysis of variance (ANOVA) test. Non-parametric variables utilized the Kruskal-Walli test. Statistical significance was set at p < 0.05. However, for group comparisons with uneven distributions of patients, adjusted p-values were ascertained by Dunn-Bonferroni adjustments to maximize statistical power and minimize the chance of a Type 1 error. Each significance level was set per study parameters. Among significantly different groups, post hoc pairwise comparisons were performed. All statistical analyses were performed in consultation with an experienced and formally trained statistician from the Northwell Health Department of Biostatistics.

## Results

**Figure 1:**
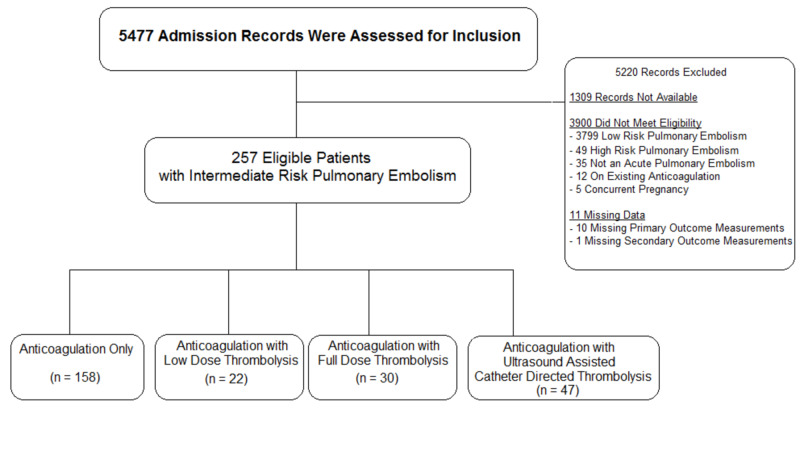
Consolidated standards of reporting trials (CONSORT) flow diagram

Out of 5,477 unique patient admissions screened over the study time, we identified 257 unique patients eligible for analysis. Unfortunately, only 11 out of 21 hospitals within the Northwell Health system had readily available administrative data to review. Further, only eight out of these 11 hospitals utilize the universal electronic medical record used for data collection. However, these eight hospitals encompassed all of the tertiary medical centres with the technical capabilities for US-CDT, intensive care unit (ICU) resources to prescribe peripherally administered thrombolytic therapy, and cardiothoracic surgery support in the event of a need for rescue therapies. Figure 1 demonstrates the participant flow diagram.

Baseline Patient Information

The baseline demographic and clinical data for the included patients, as stratified by their prescribed treatment, are demonstrated in Table 1. For initial triage disposition, 62 patients (43.7%) in the AC only group, 20 patients (91.0%) in the AC + LDT group, 28 patients (93.3%) in the AC + FDT group, and 35 patients (74.5%) in the AC + US-CDT group were initially admitted to an intensive care unit setting. There is a significant difference in rates of ICU admission among the treatment groups (P < 0.01), and post hoc analysis revealed that this difference was driven by a lower rate of ICU admission in the AC only group compared to any of the individual thrombolytic therapies. Among the four treatment groups, the baseline clinical characteristics are not significantly different. Table 2 demonstrates the baseline clinical characteristics of the study patients at presentation. A risk-stratification tier for the severity of pulmonary embolism was employed to discern different levels of disease severity in these patients to ensure equal distribution of patients among the four treatment groups. Statistical tests were conducted using Dunn-Bonferroni adjusted alpha levels of 0.005 per test. There were no significant differences between any of the presenting hemodynamic data or among the different risk stratification tiers using this alpha level. However, there were significant differences among the treatment groups regarding the modalities of confirmation of RV dysfunction. Specifically, a greater proportion of patients receiving FDT or US-CDT had RV dysfunction found on echocardiography and computed tomography than those receiving AC only or LDT. Of the three patients in the US-CDT group that started initially with low molecular weight heparin therapy, all 3 of these individuals were converted to unfractionated heparin when the decision was made to perform US-CDT.

**Table 1:** Baseline Characteristics of Study Patients For descriptive variables, quantitative data had P values calculated by ANOVA and categorical data had P values calculated by chi-square. Statistical significance set at alpha < 0.05. AC = Anticoagulation, CHF = Congestive Heart Failure, COPD = Chronic Obstructive Pulmonary Disease, FDT = Full Dose Thrombolysis, LDT = Low Dose Thrombolysis, NS = Not Significant, SD = Standard Deviation, US-CDT = Ultrasound Assisted Catheter Directed Thrombolysis , VTE = Venous Thromboembolism

	AC	AC + LDT	AC + FDT	AC + US-CDT	p Value
Number of Patients, n	158	22	30	47	
Mean age + SD, years	64.5 + 17.0	58.6 + 15.4	60.1 + 16.5	59.4 + 15.1	0.12
Male, n (%)	62 (39.2)	15 (68.2)	14 (46.7)	17 (36.2)	0.06
Race/Ethnicity, n (%)					0.24
Caucasian	91 (57.6)	8 (36.4)	18 (60.0)	33 (70.2)	
African American	48 (30.4)	8 (36.4)	10 (33.3)	13 (27.7)	
Hispanic	11 (7.0)	3 (13.6)	1 (3.3)	1 (2.1)	
Asian	2 (1.3)	1 (4.5)	1 (3.3)	0 (0)	
Mixed or Other	6 (3.8)	2 (9.1)	0 (0)	0 (0)	
Current Smoker, n (%)	8 (5.1)	2 (9.1)	1 (3.3)	5 (10.6)	0.82
History of VTE, n (%)	32 (20.3)	2 (9.1)	4 (13.3)	8 (17.0)	0.53
History of COPD, n (%)	7 (20.3)	4 (18.2)	1 (3.3)	3 (6.4)	0.07
History of CHF, n (%)	4 (4.4)	1 (4.5)	0 (0)	3 (6.4)	0.64
Active Malignancy, n (%)	26 (16.5)	4 (18.2)	0 (0)	1 (3.3)	0.09
Surgery / Trauma in past 1 month, n (%)	14 (8.9)	0 (0)	1 (3.3)	2 (4.3)	0.3
Immobilization, n (%)	11 (7.0)	2 (9.1)	0 (0)	5 (10.6)	0.34
Estrogen use, n (%)	5 (3.2)	1 (4.5)	2 (6.7)	5 (10.6)	0.23

**Table 2:** Presenting Clinical Characteristics of Study Patients P values for categorical variables were calculated using chi-square and continuous variables were calculated by Analysis of Variance. Statistical significance set at P < 0.05. AC = Anticoagulation, BPM = Beats Per Minute, BrPM = Breathes Per Minute, CHF = Congestive Heart Failure, COPD = Chronic Obstructive Pulmonary Disease, DBP = Diastolic Blood Pressure, FDT = Full Dose Thrombolysis, INR = International Normalized Ratio, LDT = Low Dose Thrombolysis, mmHg = millimeters of mercury, MN = Myocardial Necrosis, NT-proBNP = N terminal pro B type natriuretic peptide, NS = Not Statistically Significant, PT = Prothrombin Time, PTT = Partial Thromboplastin Time, RVD = Right Ventricular Dysfunction, SBP = Systolic Blood Pressure, SD = Standard Deviation, US-CDT = Ultrasound-Assisted Catheter Directed Thrombolysis, VTE = Venous Thromboembolism, PASP- Pulmonary Artery Systolic Pressure *Statistic tests were conducted using Bonferroni adjusted alpha levels of 0.005 per test. Using this alpha level, diagnostic modality confirms right ventricular dysfunction significantly differed among the four treatment groups. Post hoc tests showed AC and AC + LDT were higher than AC + FDT and AC + US-CDT

	AC	AC + LDT	AC + FDT	AC + US-CDT	p Value
SBP (mmHg), mean + SD	135.6 + 22.1	123.2 + 22.1	127.3 + 19.5	130.2 + 19.1	0.02
DBP (mmHg), mean + SD	81.9 + 14.9	81.0 + 12.9	80.1 + 14.3	81.6 + 12.3	0.93
MAP (mmHg), mean + SD	99.7 + 15.4	95.1 + 15.3	96.7 + 14.9	98.5 + 13.1	0.48
Heart Rate (BPM), mean + SD	98.8 + 19.9	111.2 + 18.2	101.5 + 20.5	101.8 + 17.1	0.05
Respiratory Rate (BrPM), mean + SD	20.1 + 5.7	22.3 + 5.3	20.9 + 7.0	21.4 + 4.7	0.25
Oxygenation Saturation (%), mean + SD	96.1 + 3.5	94.8 + 7.3	95.4 + 3.8	93.7 + 6.6	0.02
DIAGNOSTIC MODALITY, n (%)					0.21
Computed Tomographic Pulmonary Angiography	151 (95.6)	22 (100)	30 (100)	47 (100)	
Ventilation-Perfusion Scan	7 (4.4)	0	0	0	
CONFIRMATION OF RV DYSFUNCTION, n (%)					<0.001
Echocardiography	24 (15.2)*	1 (4.5)*	3 (10.0)	7 (14.9)	
Computed Tomographic Pulmonary Angiography	62 (39.2)	12 (54.5)	3 (10.0)	2 (4.3)	
Both	64 (40.5)*	8 (36.4)*	24 (80.0)	38 (80.9)	
No RV Dysfunction on Imaging	8 (5.1)	1 (4.5)	0	0	
INITIAL TREATMENT ANTICOAGULATION, n (%)					0.29
Unfractionated Heparin	121 (76.6)	21 (94.5)	25 (83.3)	44 (93.6)	
Low Molecular Weight Heparin	31 (19.6)	1 (4.5)	5 (16.7)	3 (6.4)	
Fondaparinux	1 (0.6)	0	0	0	
Argatroban	4 (2.5)	0	0	0	
Rivaroxaban	1 (0.6)	0	0	0	
RISK STRATIFICATION, n (%)					0.12
Age > 75 / Male / RVD / MN	8 (5.1)	0	1 (3.3)	2 (4.3)	
Age > 75 / Male / RVD Only	12 (7.6)	1 (4.5)	2 (6.7)	0	
Age > 75 / Male / MN Only	0	0	0	0	
Age < 75 / Male / RVD / MN	13 (8.2)	6 (27.3)	4 (13.3)	8 (17.0)	
Age < 75 / Male / RVD Only	31 (19.6)	7 (31.8)	7 (23.3)	7 (14.9)	
Age < 75 / Male / MN Only	0	1 (4.5)	0	0	
Age > 75 / Female / RVD / MN	15 (9.5)	0	2 (6.7)	5 (10.6)	
Age > 75 / Female / RVD Only	15 (9.5)	1 (4.5)	3 (10.0)	1 (2.1)	
Age > 75 / Female / MN Only	4 (2.5)	0	0	0	
Age < 75 / Female / RVD / MN	23 (14.6)	1 (4.5)	5 (16.7)	11 (23.4)	
Age < 75 / Female / RVD Only	34 (21.5)	5 (22.7)	6 (20.0)	13 (27.7)	
Age < 75 / Female / MN Only	3 (1.9)	0	0	0	
Troponin T (ng/mL), mean + SD	0.1 + 0.2	0.1 + 0.2	0.09 + 0.09	0.1 + 0.3	0.96
NT-proBNP (pg/mL), mean + SD	3326.3 + 5876.3	5180.8 + 5964.3	3129.6 + 2854.7	4217.0 + 4802.9	0.05
Initial PASP (mmHg, mean + SD)	49.3 + 18.1	51.1 + 17.9	48.6 + 18.6	55.4 + 19.0	0.1

Characteristics of Treatment in the Catheter-Directed Thrombolysis Group

All patients receiving US-CDT used unfractionated heparin for anticoagulation during the procedure. The dose of tissue plasminogen activator (tPA) administration in this group had a median value of 24mg and a median duration of 12 hours. The mean partial thromboplastin time (PTT )during the procedure of all 47 patients was 44.1 seconds. There were no significant differences between the mean PTT, mean tPA dose, or mean tPA infusion duration between the group with any bleeding and the group with no bleeding (Table 3).

**Table 3:** Treatment Characteristics of Patients Receiving Catheter Directed Thrombolysis PTT = Partial Thromboplastin Time, SD = standard deviation, tPA = Tissue Plasminogen Activator.

	Major or Minor Bleeding	No Bleeding	p Value	All Patients
	n = 3	n = 44		n = 47
Mean PTT + SD, seconds	47.7 + 4.5	43.9 + 11.7	NS	44.1 + 11.4
Mean total tPA dose administered during procedure + SD, mg	24.0 + 0	22.7 + 7.0	NS	22.8 + 6.8
Median total tPA dose administered during procedure, mg	24	24		24
Mean duration of total tPA infusion during procedure + SD , hours	12.0 + 0	12.7 + 3.1	NS	12.7 + 3.0
Median duration of total tPA infusion during procedure, hours	12	12		12

Primary Outcomes

Table 4 shows the primary and secondary outcomes for the study cohorts. Statistical significance was set at α < 0.003. All 257 of the study patients had follow-up data up to 30 days. There were 7-day mortality rates of 3.8%, 4.5%, 0%, and 0% in the AC, LDT, FDT and US-CDT groups respectively. However, this did not reach statistical significance (α =0.37). There was a statistically nonsignificant trend toward decreased 30-day mortality in both the FDT and the US-CDT groups, with rates of 8.9%, 13.6%, 0%, and 0% among the AC, LDT, FDT, and US-CDT treatment groups (α =0.04). Furthermore, there were no significant differences in the overall hospital length of stay (α = 0.301), the seven-day readmission rate (α = 0.97), or the 30-day readmission rate (α = 0.84) among the treatment groups.

**Table 4:** Outcome Data for the Study Patients p values for categorical variables were calculated using chi-square and continuous variables were calculated by Analysis of Variance.  Using Bonferroni adjusted P values, statistical significance set at α < 0.003. AC = Anticoagulation, FDT = Full Dose Thrombolysis, LDT = Low Dose Thrombolysis, ICU = Intensive Care Unit, NS = Not Statistically Significant, PE = Pulmonary Embolism, US-CDT = Ultrasound-Assisted Catheter Directed Thrombolysis a- Post hoc analyses reveals that the AC + LDT group has a significantly higher mortality rate than do the AC + FDT or the AC + US-CDT groups. Also, the AC + LDT group is non-significantly different from the AC only group. b- Because of a non-parametric distribution of data, the Kruskal-Wallis technique was instead of ANOVA. Post hoc analysis reveals only the AC only group had a lower ICU length of stay than did the other groups. c- Post hoc analysis reveals that of the patients who started in a non-ICU setting, the group treated with AC only had a reduced rate of requiring ICU upgrade.

	AC	AC + LDT	AC + FDT	AC + US-CDT	α- Value
PRIMARY OUTCOMES					
7 Day All Cause Mortality, n (%)	6 (3.8)	1 (4.5)	0 (0)	0 (0)	0.37
30 Day All Cause Mortality, n (%)	14 (8.9)	3 (13.6)	0 (0)	0 (0)	0.04
SECONDARY OUTCOMES					
1 Year All Cause Mortality, n (%)	16 (19.3)	4 (36.4)^a^	0 (0)	0 (0)	0.003
Length of ICU Stay, median days	0.5^b^	2	2	3	<0.001
Length of Hospital Stay, mean days + SD	6.5 + 4.3	8.3 + 5.4	6.7 + 2.6	6.9 + 5.9	0.31
Major Bleeding, n (%)	5 (3.2)	0 (0)	1 (3.3)	2 (4.3)	0.82
Minor Bleeding, n (%)	3 (1.9)	0 (0)	4 (13.3)	1 (2.1)	0.007
Ischemic Stroke, n (%)	0 (0)	0 (0)	0 (0)	0 (0)	N/A
Hemorrhagic Stroke, n (%)	1 (0.6)	0 (0)	1 (3.3)	1 (2.1)	0.52
Need for ICU Upgrade, n (%)	5 (5.6)^c^	2 (100.0)	2 (100.0)	10 (83.3)	0.001
Hemodynamic Instability, n (%)	6 (3.8)	4 (18.2)	2 (6.7)	1 (2.1)	0.024
Need for Mechanical Ventilation, n (%)	4 (2.5)	0 (0)	0 (0)	1 (2.1)	0.72
Need for Surgical Embolectomy, n (%)	1 (0.6)	0 (0)	0 (0)	0 (0)	0.89
Need for Catheter Thrombus Fragmentation, n (%)	1 (0.6)	0 (0)	0 (0)	0 (0)	0.89
Need for Vena Cava Interruption, n (%)	31 (19.6)	1 (4.5)	5 (16.6)	7 (14.9)	0.35
Readmitted at 7 days after discharge, n (%)	9 (5.7)	1 (4.5)	2 (6.7)	2 (4.3)	0.97
Readmitted at 30 days after discharge, n (%)	16 (10.1)	2 (9.0)	2 (6.7)	3 (6.4)	0.84
READMISSION DIAGNOSIS AT 7 DAYS, n					
Gastrointestinal Bleeding	3	0	0	0	
Intracerebral Hemorrhage	0	0	0	0	
Other major bleeding	0	0	0	0	
Worsening cardiopulmonary status due to PE	2	0	0	2	
Other	4	1	2	0	
READMISSION DIAGNOSIS AT 30 DAYS, n (%)					
Gastrointestinal Bleeding	3	0	0	0	
Intracerebral Hemorrhage	0	0	0	0	
Other major bleeding	0	0	0	0	
Worsening cardiopulmonary status due to PE	3	0	0	2	
Other	10	2	2	1	

Secondary Outcomes

There were significant differences among the treatment groups in critical secondary outcomes. All-cause mortality at one year did show a significant difference among the four treatment groups at rates of 19.3%, 36.4%, 0.0%, and 0.0% for the AC, AC+LDT, AC+FDT, and AC+US-CDT groups, respectively (α=0.003). Post-hoc analysis showed that the AC + LDT group had a significantly higher rate of 1-year all-cause mortality than either the AC + FDT group or the AC + US-CDT groups. However, there were no significant differences between the AC and the AC + LDT groups, the AC and the AC + FDT groups, or the AC and the AC + US-CDT groups. Of note, only 140 out of the initial 257 patients had one-year mortality data to review. The median length of ICU stay was significantly prolonged with the use of any thrombolytic therapy relative to AC only with 0.5 days for the AC group, two days for the AC + LDT group, two days for the AC + FDT group, and three days for the AC + US-CDT group (α < 0.001). Despite this, the mean duration of total hospital stay is not significantly different among the treatment groups (α = 0.31)

Adverse events and Safety Outcomes

There were no significantly different rates of major bleeding (α = 0.82), minor bleeding (α = 0.007), hemorrhagic stroke (α = 0.52), hemodynamic instability (α = 0.024), need for mechanical ventilation (α = 0.72), need for surgical embolectomy (α = 0.89), need for catheter thrombus fragmentation (α = 0.89), or need for vena cava interruption (α = 0.35) among treatment groups. Furthermore, there was also no difference in hospital readmission at seven and 30 days among treatment groups. Of the patients that were initially admitted to a non-ICU setting, the decision to treat with thrombolytic therapy was associated with significantly higher rates of ICU upgrade (α = 0.001). Post hoc analysis revealed that the AC only group had a significantly lower rate of requiring ICU upgrade. Of the six patients on AC only therapy who developed hemodynamic instability following prescribed therapy, two patients were initially triaged into an ICU setting, three were upgraded to an ICU setting, and one patient had passed away shortly after the onset of hemodynamic instability. None of these six patients received any thrombolytic therapies.

Risk Factor Subanalysis Among the Anticoagulation Only Group

Subgroup analysis of the anticoagulation only group is provided in Table 5. The presence of a pulmonary artery systolic pressure (PASP) > 70 mmHg on evaluation was associated with a significantly increased risk of seven-day mortality with a hazard ratio of 7.79 (95% CI 1.05 - 58.73, P = 0.048). Furthermore, the presence of an initial SBP < 130mmHg was associated with an increased risk of 30-day mortality with a hazard ratio of 23.0 (95% CI 2.92 - 180.86, P = 0.003). Finally, the presence of an initial BNP > 1400 pg/mL was associated with an increased risk of 30-day mortality with a hazard ratio of 15.33 (95% CI 1.98 - 124.37, P = 0.01).

**Table 5:** For clinically significant endpoints based upon initial presenting clinical risk factors among patients in the anticoagulation only group Statistical significance set at P < 0.05. BNP = B-type Natriuretic Peptide, DBP = Diastolic Blood Pressure, HR = Heart Rate, PASP = Pulmonary Artery Systolic Pressure, RV = Right Ventricular, SBP = Systolic Blood Pressure, Tn-I = Troponin-I, Tn-T = Troponin-T, TTE = Transthoracic Echocardiography. Myocardial necrosis is defined as initial Tn-T > 0.06 or Tn-I > 0.01.

Risk Factor	7 Day Mortality	30 Day Mortality	Hemodynamic Instability
Age > 75	2.10 (95% CI 0.41 - 10.8; P = 0.37)	1.18 (95% CI 0.38 - 3.73; P = 0.77)	1.05 (95% CI 0.19 - 5.94; P = 0.96)
Male Gender	0.77 (95% CI 0.14 - 4.32; P = 0.76)	1.17 (95% CI 0.39 - 3.58; P = 0.77)	0.77 (95% CI 0.14 - 4.32; P = 0.76)
RV Dysfunction on Both TTE and CTPA	0.73 (95% CI 0.13 - 4.09; P = 0.72)	0.80 (95% CI 0.26 - 2.51; P = 0.70)	0.73 (95% CI 0.13 - 4.09; P = 0.72)
Presence of Myocardial Necrosis	0.33 (95% CI 0.066 - 1.63; P = 0.17)	1.24 (95% CI 0.41 - 3.72; P = 0.70)	0.60 (95% CI 0.11 - 3.35; P = 0.56)
SBP < 130 mmHg	7.67 (95% CI 0.87 - 67.25; P = 0.07)	23.0 (95% CI 2.92 - 180.86; P = 0.003)	2.98 (95% CI 0.53 - 16.80; P = 0.22)
DBP < 80 mmHg	6.51 (95% CI 0.74 - 57.11; P = 0.091)	1.71 (95% CI 0.57 - 5.19; P = 0.34)	1.24 (95% CI 0.24 - 6.31; P = 0.80)
HR > 100	2.28 (95% CI 0.41 - 12.83; P = 0.35)	1.53 (95% CI 0.51 - 4.64; P = 0.45)	1.11 (95% CI 0.22 - 5.68; P = 0.90)
BNP > 1400	10.47 (95% CI 0.53 - 206.27; P = 0.12)	15.33 (95% CI 1.89 - 124.36; P = 0.01)	6.03 (95% CI 0.66 - 55.28; P = 0.11)
PASP > 50 mmHg	1.10 (95% CI 0.15 - 8.09; P = 0.92)	0.91 (95% CI 0.26 - 3.15; P = 0.88)	0.74 (95% CI 0.12 - 4.56; P = 0.74)
PASP > 70 mmHg	7.79 (95% CI 1.05 - 58.73; P = 0.048)	2.97 (95% CI 0.70 - 12.63; P = 0.14)	2.40 (95% CI 0.23 - 24.59; P = 0.46)
Risk Factor	7 Day Readmission	30 Day Readmission	Major Bleeding
Age > 75	1.74 (95% CI 0.45 - 6.76; P = 0.43)	1.29 (95% CI 0.44 - 3.78; P = 0.64)	9.02 (95% CI 0.98 - 82.9; P = 0.052)
Male Gender	0.76 (95% CI 0.18 - 3.17; P = 0.37)	0.68 (95% CI 0.22 - 2.06; P = 0.69)	2.39 (95% CI 0.39 - 14.73; P = 0.94)
RV Dysfunction on Both TTE and CTPA	0.39 (95% CI 0.78 - 1.93; P = 0.25)	0.87 (95% CI 0.30 - 2.52; P = 0.26)	0.36 (95% CI 0.039 - 3.27; P = 0.36)
Presence of Myocardial Necrosis	1.56 (95% CI 0.40 - 6.04; P = 0.52)	1.64 (95% CI 0.58 - 4.66; P = 0.35)	5.09 (95% CI 0.56 - 46.6; P = 0.15)
SBP < 130 mmHg	1.15 (95% CI 0.30 - 4.47; P = 0.84)	0.84 (95% CI 0.29 - 2.45; P = 0.76)	0.35 (95% CI 0.038 - 3.18; P = 0.35)
DBP < 80 mmHg	1.57 (95% CI 0.41 - 6.09; P = 0.51)	1.25 (95% CI 0.45 - 3.53; P = 0.67)	0.81 (95% CI 0.13 - 5.00; P = 0.82)
HR > 100	0.88 (95% CI 0.23 - 3.40; P = 0.85)	0.47 (95% CI 0.15 - 1.41; P = 0.18)	0.27 (95% CI 0.029 - 2.44; P = 0.24)
BNP > 1400	4.63 (95% CI 0.90 - 23.70; P = 0.07)	2.42 (95% CI 0.81 - 7.17; P = 0.11)	0.47 (95% CI 0.048 - 4.61; P = 0.52)
PASP > 50 mmHg	0.34 (95% CI 0.067 - 1.78; P = 0.20)	0.66 (95% CI 0.20 - 2.14; P = 0.49)	0.26 (95% CI 0.03 - 2.42; P = 0.24)
PASP > 70 mmHg	0.37 (95% CI 0.02 - 6.70; P = 0.50)	0.20 (95% CI 0.01 - 3.68; P = 0.28)	0.59 (95% CI 0.031 - 11.11; P = 0.72)

## Discussion

In our retrospective review, there was no significant difference in the primary outcomes of seven or 30-day mortality in a cohort of patients with intermediate-risk pulmonary embolism treated with full anticoagulation, anticoagulation in combination with full and half-dose thrombolytic, and anticoagulation plus ultrasound-assisted catheter-directed thrombolysis. There was, however, a significant decrease in all-cause mortality at one year in favour of both the catheter-directed and full-dose thrombolytic groups compared to the use of low dose thrombolysis. One may hypothesize that this increased mortality with low dose thrombolysis was due to RV failure, but our study was not powered to assess this. Given the positive findings for one-year mortality, and the subgroup analysis of the anticoagulation only group, the next logical question would be whether patients with initial echocardiographic evidence of pulmonary artery systolic pressure of > 70, SBP < 130, or those who have elevated BNP on admission may do better with full-dose or catheter-directed thrombolytic, while patients who lack these high-risk features may do well with standard anticoagulation alone. Our study results report on hard clinical endpoints that add new perspectives on the utilization of thrombolytic therapy in patients with pulmonary embolism that have not been previously reported in the literature. The four treatment groups were well-matched in terms of clinically significant baseline characteristics with few exceptions. Our use of a risk stratification scheme was vital in ensuring a similar degree of illness acuity was present in the compared groups. Further, using Dunn-Bonferroni adjusted p-values (α-values) enabled an extra degree of statistical rigour that also helped ensure our data's valid interpretation.

The higher mortality at one year in the low-dose thrombolytic group argues against this approach in intermediate-risk pulmonary embolism, at least until a well-designed clinical trial confirms these results. The MOPETT trial, the major positive trial in favour of low-dose thrombolysis, utilized the development of pulmonary hypertension or the composite endpoint of pulmonary hypertension and recurrent PE as primary endpoints. MORPHETT also evaluated the secondary endpoints of total mortality, duration of hospital stay, and bleeding. At 28 months of follow up, there was a significant decrease in pulmonary hypertension and recurrent pulmonary embolism in the low dose thrombolytic group but no difference in mortality. Our results show discordance with the MOPETT trial regarding mortality, as our cohort who got low dose alteplase had a significantly increased mortality rate at one year. Whether a reduction in pulmonary artery pressures will definitively lead to a reduction in long-term mortality is unknown. In MOPETT, Sharifi et al. did show that half-dose alteplase led to a mean decrease of 21mmHg in the PASP compared to anticoagulation alone [3]. 

There are substantial data that show thrombolytic therapy can reduce pulmonary artery pressures. However, only two randomized controlled trials have demonstrated complex clinical outcomes using thrombolysis in the intermediate-risk PE population. Konstantinides et al., a randomized controlled trial of standard anticoagulation versus anticoagulation plus full-dose alteplase, demonstrated a significantly reduced composite outcome of 30-day all-cause mortality and escalation of therapy [2]. The PEITHO trial, a randomized controlled trial of standard anticoagulation versus anticoagulation plus full-dose tenecteplase, demonstrated a statistically significant decrease in the composite outcome of death or hemodynamic decompensation at seven days in the tenecteplase arm [9]. This was, however, primarily driven by a decrease in hemodynamic decompensation and came at the expense of a ten-fold increased risk of hemorrhagic stroke and a greater than five-fold increased risk of major extracranial bleeding. Both studies did not yield any statistically significant differences when the mortality rates were assessed independently in both studies. The ULTIMA trial was a randomized controlled trial that demonstrated that US-CDT significantly reduced pulmonary pressures than heparin therapy alone (mean pulmonary artery pressure 30mmHg to 24mmHg and PASP from 51mmHg to 38mmHg) [10]. Several uncontrolled studies have also been performed subsequently and have yielded results similar and consistent with those in the ULTIMA trial [6,11-16]. However, none of these was powered to assess mortality. Peripherally administered thrombolysis (full dose or low dose) has also yielded similar magnitudes of pulmonary artery pressure reductions. In a multicenter randomized controlled trial, Meyer et al. demonstrated that the use of 100mg of alteplase for high-risk pulmonary embolism yielded a mean decrease in mean pulmonary artery pressure (mPAP) by 11mmHg [17]. Meneveau et al. and Tebbe et al. followed up with multicenter randomized controlled trials that found that 100mg of alteplase for high-risk pulmonary embolism yielded a mean decrease in mPAP 14mmHg and 10mmHg, respectively [18,19]. Similarly, Mi et al. also showed that the use of half-dose tPA (50mg IVPB over 2 hours) reduced mean PASP by 21mmHg compared to anticoagulation alone [20]. Despite the advantages of hemodynamic improvement, the data on mortality reduction remains scarce.

Our subgroup analysis on the AC only group supports the notion that patients with severe pulmonary hypertension, PASP > 70 mmHg, are at higher risk of mortality within the first seven days if maintained on anticoagulation alone. Other studies have echoed these observations. Trujillo-Santos et al. published a meta-analysis of 3,395 patients with PE and RV dysfunction on CTPA, measured by an RV/LV ratio had demonstrated an at least 2-fold increased risk of PE-related mortality with a dilated RV [21]. He suggested that CT-assessed RV dilation yielded an odds ratio for mortality of 1.8. However, CT provides a static measure while echocardiographic pulmonary artery pressures can be measured frequently in real-time with a point of care echocardiography [22]. Two subsequent systematic reviews and meta-analyses in cohorts of normotensive patients with PE also suggested a significantly increased risk of mortality with RV dysfunction. We, therefore, propose classifying intermediate-risk PE into subdivisions based on PA pressure above or below 70 mmHg. Such an approach would require an experienced and skilled echocardiographer to be present at or near the point of care decision making for these patients in the emergency room. While there is no evidence currently to support treating intermediate-risk PE with PASP > 70mm Hg differently, we would suggest that at the very least, these patients be triaged to the ICU, as opposed to intermediate care, given the significant association with seven-day mortality.

A statistically significant decrease in all-cause mortality at one year favoured the catheter-directed and full-dose thrombolytic group. This should be interpreted with caution, however. One possible reason for this is lower pulmonary hypertension and cor pulmonale in these treatment groups. However, our study was not powered to detect this difference. Other factors could have been responsible for the patients' lower mortality rates one year out. A future randomized controlled trial should include long-term follow up of the patients in the various arms for long term mortality and development of pulmonary hypertension.

The retrospective nature of our study demands that caution be exercised from making definitive conclusions in light of the possibility of confounding and selection bias. The patients diagnosed with intermediate-risk PE at a non-tertiary care hospital may not have received consideration for transfer for fibrinolytic therapies. Depending on which speciality (cardiology, cardiothoracic surgery, critical care, or pulmonology) was assigned to the pulmonary embolism response team for a given patient on a given day, it is possible that the selection bias of the treating team would have favoured a particular treatment. Further, our study could only identify all-cause mortality as determined by the date of expiration in the medical records. However, the precise cause of death for all patients cannot be determined from our chart review. Therefore, it is not possible to identify the role that confounders may have had on the outcomes. In-hospital mortality rates were lower in our study (2.7%, as represented by the 7-day mortality rate of all included patients) than the mortality rate of intermediate-risk PE patients in the analysis of the Management Strategies and Prognosis of Pulmonary Embolism Registry (which shows an 8% in-hospital mortality rate) [23]. However, improved patient care techniques and technologies have decreased mortality rates over the past two decades. Due to a dearth of follow up echocardiographic tests performed for most of these patients, we could not report the change in PASP parameters after their treatment interventions. This limits our ability to attribute any improvement in PASP to the positive hemodynamic effects of thrombolytic therapy, which has been shown in other studies.

We believe our findings support the approach favoured by the current ACCP guidelines. Our data show a lack of mortality reduction and increased ICU and hospital resource use associated with the use of thrombolytic therapies compared to using anticoagulation alone in a population of patients diagnosed with intermediate-risk pulmonary embolism. Although the use of FDT or US-CDT is associated with a significantly more significant reduction in 1-year all-cause mortality, the role of potential confounders and the incomplete patient mortality data for the cohort at one year preclude adequate comparison. Based on our data, low dose thrombolytics should be avoided.

## Conclusions

The addition of systemic and catheter-directed thrombolytic therapy was not associated with a significant improvement in seven or 30-day mortality rates. It was associated with greater ICU length of stay in a cohort of patients with intermediate-risk PE. Thus, our f.indings support using the current ACCP recommendation of initial anticoagulation alone to treat intermediate-risk pulmonary embolism.
